# Using Three-Dimensional Printing Technology to Solve Complex Primary Total Hip Arthroplasty Cases: Do We Really Need Custom-Made Guides and Templates? A Critical Systematic Review on the Available Evidence

**DOI:** 10.3390/jcm13020474

**Published:** 2024-01-15

**Authors:** Giuseppe Anzillotti, Edoardo Guazzoni, Pietro Conte, Vincenzo Di Matteo, Elizaveta Kon, Guido Grappiolo, Mattia Loppini

**Affiliations:** 1 IRCCS Humanitas Research Hospital, Via Manzoni 56, Rozzano, 20089 Milan, Italy; doc.anzillotti@gmail.com (G.A.); e.guazzoni@gmail.com (E.G.); pietro.conte@humanitas.it (P.C.); drvincenzodimatteo@gmail.com (V.D.M.); elizaveta.kon@humanitas.it (E.K.); guido.grappiolo@mac.com (G.G.); 2 Department of Biomedical Sciences, Humanitas University, Via Rita Levi Montalcini 4, Pieve Emanuele, 20072 Milan, Italy; 3 Faculty of Medicine and Surgery, Catholic University of Sacred Heart, Largo Francesco Vito 1, 00168 Rome, Italy; 4 Department of Traumatology, Orthopaedics and Disaster Surgery, Sechenov University, Moscow 119991, Russia; 5 Fondazione Livio Sciutto Onlus, Campus Savona, Università Degli Studi di Genova, 17100 Savona, Italy

**Keywords:** 3D printing, hip, total hip arthroplasty, development dysplasia hip

## Abstract

The burden of osteoarthritis (OA) is around 300 million people affected worldwide, with the hip representing a commonly affected joint. Total hip arthroplasty (THA) has been used with notable success as a definitive treatment to improve pain and function in hip OA patients. The recent advent of new technologies, such as 3D printing, has pushed the application of these new concepts toward applications for the well-known THA. Currently, the evidence on the use of 3D printing to aid complex primary THA cases is still scarce. Methods: An extensive literature review was conducted to retrieve all articles centered on the use of 3D printing in the setting of primary THA. Results: A total of seven studies were included in the present systematic review. Four studies investigated the use of 3D-printed surgical guides to be used during surgery. The remaining three studies investigated the benefit of the use of 3D-printed templates of the pelvis to simulate the surgery. Conclusions: The use of 3D printing could be a promising aid to solve difficult primary total hip arthroplasty cases. However, the general enthusiasm in the field is not supported by high-quality studies, hence preventing us from currently recommending its application in everyday practice.

## 1. Introduction

On a worldwide scale, up to 300 million people are affected by osteoarthritis (OA), with the hip representing one of the most involved joints [1]. The pain is just the tip of the constellation of OA consequences, which include the almost unbearable social and economic costs, which are estimated to be around 303 billion yearly, both due to healthcare expenses and loss of profit [2,3,4]. Since its introduction in the 1960s [5], total hip arthroplasty (THA) has proven its effectiveness in reducing symptoms and improving function, hence leading to an overall improvement in quality of life and reduction of healthcare service usage [6,7]. In order to ensure the longest implant survival and avoid reoperation procedures, several studies and approaches [8,9] were developed, considering the osteointegration of the prosthetic materials, biomaterial wear, and the prosthetic component positioning, thus ensuring the hip biomechanics [10,11,12]. Joint replacement surgery, with its focus on implants, instruments, and surgical devices, is well suited for the application of three-dimensional (3D) printing: a process of design and manufacturing and layer-by-layer construction of anatomically detailed models and surgical guides, with the promise to revolutionize medicine and healthcare [13]. In the operating room, 3D printers are working to assist the orthopedic surgeon both intraoperatively, through cutting guides facilitating crucial surgical steps, and even before surgery, via 3D templates better reflecting each patient’s peculiar anatomy [14,15,16]. Previous studies also assessed its efficacy in decreasing the operative time, blood and bone loss, and trauma for the patient [17,18]. In fact, the use of 3D printing templates is able to provide the surgeon with more information on patient-specific anatomy that can be hands-on investigated prior to surgery in complex procedures, hopefully giving a better understanding than the conventional two-dimensional (2D) radiographic reconstructions [19]. The adoption of this new perioperative asset has been recently investigated in the setting of total hip and knee revision arthroplasties, reporting satisfactory clinical and radiological outcomes [20]. However, only a few studies investigated 3D printing, intended both as templates and guiding devices, to assist complex primary THA. Such technologies have been proposed as an additional aid in the setting of complex cases of the hip undergoing THA. Furthermore, 3D-printed templates of the patient’s pelvic anatomy are intended to be used during a prior surgical simulation to better plan the THA procedure, whereas 3D-printed cutting guides have been developed to be used during surgery to improve the accuracy of both the acetabular cup and femoral stem placement. Hence, the aim of the present systematic review is to summarize the existing literature on the application of such 3D-printed technologies in complex primary THA, evaluating the potential benefits and eventual disadvantages.

## 2. Materials and Methods

Literature research was performed on PubMed, Embase, and Google Scholar databases on the 23 September 2023, utilizing the following search string: “3D printing” OR “three dimensional printing” OR “3D assisted” OR “3D guides” OR “three dimensional guides” OR “printed template” AND “primary total hip arthroplasty” OR “primary THA” OR “primary total hip replacement” OR “primary THR”. The screening process was performed by two independent reviewers (G.A. and P.C.). The first step was the initial screening based on titles and articles, considering the following inclusion criteria: (1) randomized controlled trials (RCTs); retrospective or prospective studies on humans; (2) English language; (3) published in indexed journals; and (4) evaluating the outcomes of 3D printing technology for difficult primary total hip arthroplasties. Exclusion criteria were articles written in other languages, pre-clinical studies, reviews and meta-analyses, and studies on the use of 3D printing for different procedures. Furthermore, this study was focused on 3D-printed technologies that help surgeons in complex THA procedures; therefore, reports on 3D-printed custom-made implantable prostheses have not been included.

**Figure 1:**
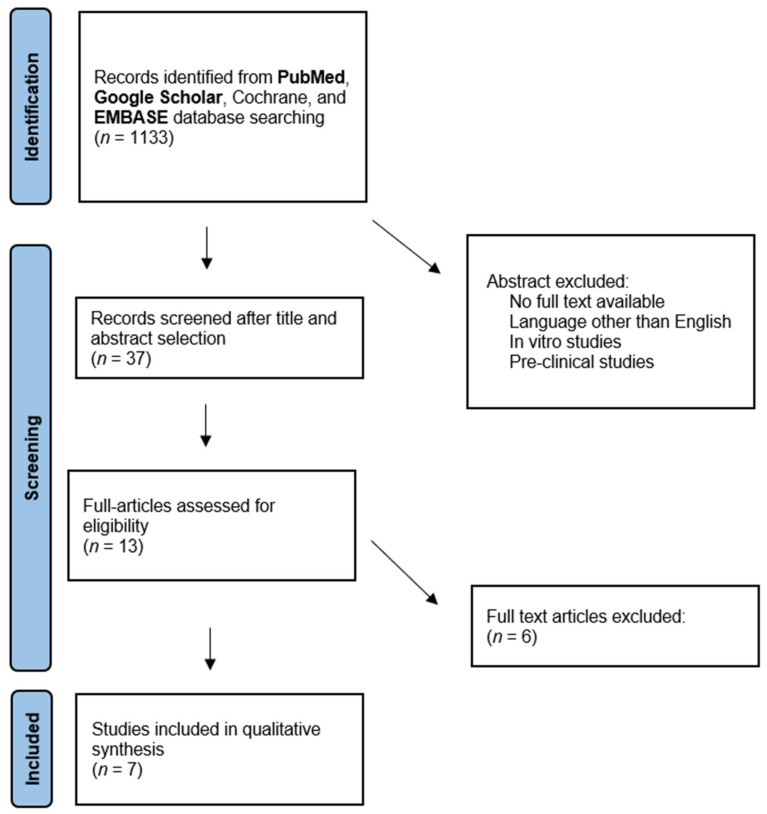
PRISMA (Preferred Reporting Items for Systematic Review and Meta-Analysis) flowchart of the systematic literature review.

Upon concluding the initial screening phase, full texts of included articles were evaluated, and the reference list of all the retrieved articles was further evaluated for identification of potentially relevant studies. A PRISMA flowchart of the selection process is reported in Figure 1.

Discrepancies encountered between the two reviewers were solved by a senior investigator (M.L.). The full texts included underwent data extraction and subsequent collection for the purposes of the present project.

## 3. Results

A total of seven studies were included in the present systematic review. Four studies [21,22,23,24] investigated the use of 3D-printed surgical guides: in all four studies, a preoperative CT scan of the pelvis was performed, and, with the aid of specific software, 3D surgical guides were designed and printed. Those guides were then sterilized and temporarily fixed to the patient’s pelvis during THA surgery to guide the surgeon and to improve the accuracy of acetabular cup placement (all four studies previously mentioned) or even femoral osteotomy [22] in primary complex THA.

The remaining three studies investigated the benefit of the use of 3D-printed templates of the pelvis in complex primary THA [18,25,26]. In all three studies, a CT scan was conducted, and an entire 3D-printed template of the specific patient’s pelvis was produced to perform a pre-surgery simulation of the THA procedure. The aim of producing those real-size anatomical templates was to better plan the cup positioning and to more precisely estimate cup dimensions and the eventual need for additional specific surgical devices such as augments and contouring plates. Compared to surgical guides, templates were not used during surgery but exclusively during the preoperative simulation.

### 3.1. Quality Assessment of the Retrieved Studies

For randomized controlled trials (RCTs), the risk of bias was assessed using the Cochrane Risk of Bias Tool 2 for Randomized Controlled Trials (RoB 2 tool) [27]. Seven types of bias were analyzed and classified into “low risk”, “high risk”, or “unclear risk”. For both the studies analyzed, the randomization process and the allocation concealment method were not sufficiently described, leading to some concerns in the overall judgment (Table 1). Then, the results of this evaluation were converted to Agency for Healthcare Research and Quality standards, which ultimately ranked the trials as “good quality”, “fair quality”, and “poor quality”. None of the randomized controlled trials included in the present systematic review reached a standard of “good quality”, and both the RCTs reached a standard of “fair quality”.

For studies with a non-randomized design, the risk of bias was carried on through the ROBINS-I tool (“Risk Of Bias In Non-randomised Studies-of Interventions”) [28], which evaluates the eventual benefit or harm of an intervention in studies that did not use the randomization process. (Table 2). Two studies reported a “moderate” overall risk of bias [25,26], and one study reported a “serious” risk of bias [24], mainly caused by participants’ selection and outcomes’ measurements.

### 3.2. 3D-Printed Surgical Guides

A synopsis of the included studies evaluating 3D printing in the setting of the development of intraoperative guides is reported in Table 3.

Among the four studies investigating the use of surgical jigs, two presented a randomized design [21,23]: one was a prospective trial [22], and one was a case–control study [24].

Yan et al. [21] published an RCT comparing 12 THA performed with the 3D-printed navigation templates for the acetabulum versus 13 THA with the standard technique in patients affected by OA secondary to developmental dysplasia of the hip (DDH). A Computer Tomography (CT) scan was utilized to evaluate the positioning of the implants. They found a statistically significant reduction in operative time, intra-operative bleeding, and postoperative hemorrhage and a statistically significant increase in the Harris Hip Score (HHS) at 6 months in the 3D-printed group. They also found no statistically significant difference in abduction and anteversion angle and distance between the center of rotation (COR) and the ischial tuberosity from the affected side and the prosthesis in the 3D-printed group, while the anteversion angle and the distance were larger in the control group.

Mishra et al. [23] evaluated the difference in cup placement between two groups in their RCT (18 hips each); one group operated with the aid of a 3D-printed jig to help in the cup reaming and positioning, and the other operated with the standard technique. They evaluated the results with postoperative X-rays, and in the 3D-printed jig group, the anteversion angles were significantly closer to their proposed safe zone compared to the other one. They did not find any statistically significant difference in surgical time, blood loss, surgical time for cup placement, or cup abduction angle.

Tu et al. [22] evaluated the accuracy and the results of the use of 3D-printed jigs for acetabular cup placement and femoral shortening osteotomy for the treatment of Crowe type IV DDH. This was a prospective study on 12 patients (12 hips) with an average follow-up of 72.42 months (38–135 months). The authors found that the guiding template faithfully matched the bony landmark of the acetabulum and proximal femur. The HHS improved from 34.2 ± 3.7 preoperatively to 85.2 ± 4.2 postoperatively. Leg length discrepancy decreased from 51.5 ± 6.5 mm preoperatively to 10.2 ± 1.5 mm postoperatively. The visual analog scale for pain score decreased from 6.2 ± 0.8 preoperatively to 1.3 ± 0.3 postoperatively. They reported one dislocation 2 weeks after surgery and one sciatic nerve palsy, both resolved without surgery.

Hananouchi et al. [24] investigated the accuracy of the cup placement with and without the use of a 3D-printed guide. The authors divided the patients into two groups: one operated with a standard technique (38 hips) and one with a surgical guide (31 hips). Afterward, they evaluated the number of outliers, defined as a cup placed beyond 10° from preoperatively planned alignment. All of the patients had a preoperative CT scan for surgical planning and a postoperative CT scan to evaluate the cup orientation. The use of the surgical guide reduced the number of outliers (0%; zero out of 31 cases) compared with the group operated with the standard technique (23.7%; 9 out of 38 cases). This result was achieved with no difference in operative time (*p* = 0.06) or blood loss (*p* = 0.73) between the two groups.

Furthermore, among the studies using surgical jigs, both resin and polylactic acid were used to realize the model, with variable costs, reported in detail in Table 4.

### 3.3. 3D-Printed Models for Surgical Simulations

A synopsis of the included studies assessing the use of 3D printing to produce surgical phantoms is reported in Table 5.

Of the three studies investigating the benefit of the use of a 3D-printed model of the pelvis to simulate the surgery, one was a retrospective study [26], one was a prospective study [25], and the last one was a pilot study with surgical simulation.

Zhang et al. [26] investigated the use of a 3D-printed model in 21 difficult primary THA (17 patients) and evaluated their ability to assess the bone defect size and the clinical and radiological outcomes. The bone defect evaluation shows no statistically significant difference between the sizes of bone defects in the 3D-printed model and during surgery (4.58 ± 2.47 cm^2^ in the simulation and 4.55 ± 2.57 cm^2^ in the surgery; t = 0.03, *p* = 0.97). There was a high rate of agreement between the size of the cup preoperatively planned and the one implanted (ICC = 0.93). The mean vertical and horizontal distances of the hip rotation center on the pelvic radiographs were restored to 15.12 ± 1.25 mm and 32.49 ± 2.83 mm, respectively. Xu et al. [25] published a prospective study on 10 patients (14 hips) who underwent THA with the aid of a 3D-printed model for OA due to DDH. The mean follow-up was 23.1 ± 5.9 (14–30) months. The mean HHS at the last examination was 83.3 ± 5.7 (pre-op: 37.7 ± 6.8). No perceptible limb length discrepancy (LLD) was reported after a six-month follow-up. The authors evaluated the position and the bone coverage of the cups on the X-rays showing a mean abduction angle of 45.1° (40.2°–53.5°), a mean horizontal location of the hip center from the teardrop of 21.7 mm (15.0–31.2 mm), a mean height of the hip center from the inter-teardrop baseline of 18.8 mm (11.5–25.8 mm), and at least 80% of the cup contained by bone in each case. At the last follow-up, no implants showed signs of mobilization. There was a higher rate of agreement with the size of the cup preoperatively planned with the 3D simulation than the one planned with the 2D template. The difference in excellence rate (a difference of ≤two sizes) in the prediction of the prosthesis between 3D preoperative planning and 2D template measuring method was of statistical significance (χ^2^ = 8.023, *p*= 0.05). Jiang et al. [18] published a pilot study investigating the usefulness of 3D-printed models and the properties of different materials in simulated preoperative surgery and compared them with preoperative planning based on 2D CT scans and X-ray imaging. Seven patients (seven hips) with complex anatomy were enrolled, and patient-specific models were 3D-printed in plaster, resin, and nylon. Resin models provided the most realistic trials of implant impaction; conversely, nylon models underwent rapid bony distortion under reaming. In conclusion, the authors referred to a superior clinical, logistical, and educational outcome when using the 3D model planning compared to the 2D CT scan and X-ray imaging model planning.

Costs and materials used to produce a surgical model for preoperative simulation are reported in Table 4.

## 4. Discussion

The main finding of the present systematic review is that 3D printing offers no disadvantages in the setting of complex primary total hip arthroplasty cases. The investigation of clinical and radiological superiority of this new technique over traditional surgery is undermined by the wide heterogeneity of the studies, including notable differences in the baseline condition (DDH, Perthes disease, OA, and pelvic fractures), material used, and outcome measures assessed.

The introduction of 3D printing in the context of the surgical field arises as an interconnected mixture of modern digital capture technology, computer-aided design, numerical control technology, laser or electron beam technology, and the most innovative materials all being integrated [29]. Significant recent breakthroughs have been made in 3D printing technology and associated software [30]. In the medical industry, a combination of 3D printing and CT scanning technology allows for the capture of a digital grid model in biological forms to 3D print a corresponding physical model with the same shape and internal structure as the biological anatomical part [31]. A new age has begun with the development of 3D printing, which makes it possible to convert patient-specific imaging data into realistic three-dimensional models that the surgeon can evaluate with hands-on experience prior to surgery. As a result, the use of 3D printing technology in medical reconstruction has given rise to a new discipline known as digital medicine [32]. Indeed, orthopedic surgeons can now reach previously unachievable insights into each patient’s distinct anatomy, enabling a thorough comprehension that goes beyond the limitations of conventional planning techniques. Surgeons may now examine, explore, and understand the patient’s hip joint in three dimensions, which paves the way for a paradigm change. Since the development of this new technology, limitless potential has been perceived in orthopedics, especially in the field of joint replacements. The systematic review conducted by Zhang et al. [20] analyzed the outcomes obtained by ten studies that used 3D printing-assisted surgery for revision total hip and knee arthroplasty. Only two out of ten studies examined reported the presence of a control group; nonetheless, the authors conclude that this technology can offer satisfactory radiological and clinical outcomes. Conversely, we retrieved only articles focused on the use of 3D printing to assist primary THA, and only three [21,23,24] of them had a control group. Moreover, despite all the authors adopting this new technique for complex cases, we realized that there is no univocal definition of a complex primary THA since it is usually an operator-related definition rather than radiologically dependent. However, many of the authors tend to use this adjective to outline the most advanced cases of developmental dysplasia of the hip (DDH), rated according to the Crowe classification [33]. Five [18,21,22,25,26] out of the seven studies included considered the advanced DDH as a criterion able to increase the surgical complexity, in particular, related to the cup positioning. Moreover, we made a distinction between 3D printing to produce surgical guides and 3D printing to produce surgical models. Not unexpectedly, the studies with a randomized design belong to the first group; however, the patients included may appear quite diverse. Yan et al. [21] included patients affected by DDH Crow II-IV; conversely, Mirsha et al. [23] included patients with no further specified complex anatomy, leading to a major concern regarding comparisons between the two studies. In fact, the former [21] reports superior outcomes when 3D-printed surgical guides are used, while the latter [23] does not assess such differences, reporting comparable inter-group results. Furthermore, they even reported different outcomes, including cup anteversion, a concept that has to be integrated with the combined anteversion and the pelvic tilt [34,35], making the radiological comparison even more challenging.

Furthermore, the reduction in surgical times is one of the most common outcomes assessed in the included studies. In fact, one of the most promising applications of 3D-printed aided surgery is the shortening of surgery. This aspect is ultimately related to the consequent reduction in the occurrence of postoperative infection. With an incidence rate ranging between 0.4% and 1.4% [36,37,38], the corollary of a periprosthetic hip infection can deeply impact both the patient’s life and the healthcare system. Indeed, the 5-year survival after a diagnosis of periprosthetic hip infection is even lower than common malignancies such as melanoma, breast cancer, and other common tumors [39], with outrageous annual direct and indirect costs, estimated to be up to USD 753.4 million annually by 2030 [40]. Hence, we believe that each effort is fundamental to reducing the occurrence rate of this disastrous complication. Among the studies included in the present systematic review, only the RCT by Yan et al. [21] reported a decreased surgical duration compared to conventional surgery. None of the other studies adopting the 3D printing surgical guides reported significant differences. Furthermore, none of the studies evaluating the use of 3D-printed templates mentioned eventual variations in surgical times compared to conventional surgery. Despite that, we can hypothesize that exact knowledge of a patient’s three-dimensional anatomy, assessed by the surgeon’s hand and instruments, would significantly shorten the surgery time compared to a simple assessment of bidimensional radiological imaging. However, the lack of a control group makes this assumption a mere opinion of the authors based on their own experience in this field.

Nonetheless, even if the customization and the advent of tailored medicine, built on the patient, for the patient, make the use of 3D printing a cutting-edge piece of technology for each orthopedic surgeon, at the end of the day, a cost–benefit analysis appears necessary. The cost of each surgical case ranged from USD 4 to USD 1500; however, most of the included studies did not account for the additional indirect expenses due to the preoperative CT scan and outsourcing of the procedure. Moreover, different prices are mostly a result of the type of material used, and we do not exactly have sufficient data to assess if the material characteristics are comparable [18]. Hence, the ability to strike a balance between therapeutic benefits and budgetary considerations is critical for the long-term incorporation of this technology into everyday practice.

However, we are well aware that these conclusions should be considered only as a photograph of the available evidence and taken as a bouquet of indications for future research rather than a definitive judgment. As shown in the risk of bias assessment of the retrieved studies (Table 1 and Table 2), the quality of the current data is still insufficient to provide categorical recommendations for the application of 3D printing in the fields analyzed.

However, we believe that the major strength of our study is the novelty of the topic examined and the collection of all the available evidence in such a limited research corner. However, the present systematic review is not free from limitations. Firstly, the broadened definition of ‘complex’ primary THA is not scientifically accepted and includes a wide variety of conditions, anatomies, and patients, hence diluting any scientific consideration. Specifically, a wide variety of conditions were found to be considered ‘complex’ cases in the included studies, spanning from Perthes disease to DDH, pelvic fractures, and advanced primary hip OA. The different types of 3D printers used and the different materials contribute to the addition of further variability to our findings. Furthermore, a major burden of the current evidence is represented by the often inadequate methodological quality of the included studies, which is particularly undermined by the lack of high-quality RCTs, inadequate patient selection, and diverse outcome measures reported. Moreover, the exiguous number of both patients and studies included leads us to believe we are still in the learning curve phase of this new era, maybe in a premature time to draw hasty conclusions.

## 5. Conclusions

The use of 3D printing could be a promising aid to solve complex primary THA surgeries, but the current evidence is undermined by the small number of cases, different materials and techniques, and diverse outcomes assessed. Hence, the lack of high-quality studies does not justify the general enthusiasm in the field, preventing us from currently recommending its application in everyday practice.

## Figures and Tables

**Table 1:** Quality assessment for the included RCTs using the Cochrane RoB 2 tool.

		Risk of Bias Domains					
		D1	D2	D3	D4	D5	Overall
Study	Yan et al. (2020) [21]	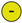	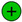	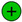	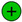	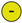	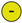
	Mishra et al. (2020) [23]	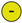	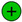	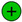	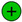	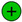	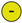

Domains: D1: Bias arising from the randomization process. D2: Bias due to deviations from intended intervention. D3: Bias due to missing outcome data. D4: Bias in measurement of the outcome. D5: Bias in selection of the reported result. Judgement: 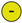, Some concerns; 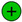, Low.

**Table 2:** ROBINS-I tool for the risk of bias assessment of the non-RCTs included studies.

Author (Year Pub)	Baseline Confounding	Selection of Participants	Classification of Intervention	Deviation from Intended Intervention	Missing Data	Measurement of Outcomes	Selection of Reported Results	Overall Risk of Bias
Tu et al. [22] (2020)	Low	Low	Low	Low	No information	Low	Low	Low
Hananouchi et al. [24] (2010)	Moderate	Moderate	Moderate	Low	Low	Moderate	Moderate	Serious
Zhang et al. [26] (2021)	Low	Low	Low	Low	No information	Moderate	Low	Moderate
Jiang et al. [18] (2021)	Moderate	Serious	Low	Low	No information	Low	Moderate	Low
Xu et al. [25] (2015)	Low	Moderate	Low	Low	No information	Low	Moderate	Moderate

**Table 3:** Synopsis of the studies focused on the use of 3D-printed jigs.

Author (Year)	Design	Number of Patients	Type of 3D-Printed Device	Disease	Follow-Up	Outcome Measures	Results
Yan et al. [21] 2020	RCT	12 with 3D-printed guides vs. 13 without	PLA 3D-printed acetabular guiding	DDH (Crowe II–IV)	1.6 years (1.2–3.8)	Operation time, intraoperative hemorrhage, postoperative drainage, infection, loosening, HHS acetabular position with CT scan, distance from COR to ischial tuberosity	Lower operation time, blood loss *, and higher HHS at 6 months in the 3D-printed group. More precise replication of contralateral acetabular angles in the 3D-printed group
Tu et al. [22] 2020	Prospective	12	Resin 3D-printed guiding template for cup position and femoral osteotomy	DDH (Crowe IV)	72.42 months (38–135)	HHS, leg length discrepancy, visual analog scale scores	Improvements in HHS *, LLD *, and VAS *
Mishra et al. [23] 2020	RCT	36: 18 3D-aided vs. 18 conventional cup placement	PLA 3D-printed acetabular jig for guiding cup placement	Complex anatomy	-	Blood loss, total surgical duration, surgical duration of cup placementCup angle of inclination, angle of anteversion, differences in acetabular offset and hip length	No significant differences in blood loss and surgical timings No significant differences in accuracy of cup placement but less variability and outliers in 3D-aided group
Hananouchi et al. [24] 2010	Case–control	31 with surgical guides vs. 38 without	Resin 3D-printed surgical guide	OA	-	Alignment accuracy, operating time, total blood loss	The surgical guide provided more reliable cup insertion compared with conventional techniques. No differences in total blood loss or operating time

RCT: randomized controlled trial; DDH: developmental dysplasia of the hip; COR: center of rotation; OA: osteoarthritis; HHS: Harris Hip Score; LLD: leg length discrepancy; CT: Computer Tomography; PLA: polylactic acid; VAS: visual analog scale; * = *p* < 0.05.

**Table 4:** Material and costs of the 3D equipment used.

Author (Year)	Material	Cost of Software	Costs Cost of the 3D Printer	Cost of Material/Case
Yan et al. [21] 2020	PLA 3D-printed acetabular guides	-	-	-
Tu et al. [22] 2020	Resin 3D-printed guiding template for cup positioning and femoral osteotomy	-	-	USD 100
Mishra et al. [23] 2020	PLA 3D-printed acetabular jig for cup placement	-	-	USD 4–USD 6
Hananouchi et al. [24] 2010	Resin 3D-printed surgical guide	USD 15,000–USD 30,000	USD 120,000	USD 50–USD 100
Zhang et al. [26] 2021	PLA life-sized 3D-printed template for surgical simulation	-	-	-
Jiang et al. [18] 2021	Plaster, resin, and nylon 3D-printed acetabular models	Outsourced	Outsourced	Plaster: USD 200 Resin: USD 1500 Nylon: USD 100
Xu et al. [25] 2015	3D-printed model for preoperative surgical simulation made of fluid-binding substances and ink	-	-	USD 400

PLA: polylactic acid.

**Table 5:** List of the studies using 3D models for surgical simulation.

Author (Year)	Design	Number of Patients	Type of 3D-Printed Device	Disease	Follow-Up	Outcome Measures	Results
Zhang et al. [26] 2021	Retrospective	17 patients/21 hips	PLA life-sized 3D-printed template for surgical simulation	DDH	18.35 ± 6.86 months	Preoperative and postoperative hip rotation center data measurement on pelvis plain films, bone defect area, and HHS	High rate of accordance in the sizes of the acetabular component and the bone defect between preoperative planning on the 3D print model and THA. Improvement of HHS *
Jiang et al. [18] 2021	Pilot Study with Surgical Simulation	7	Plaster, resin, and nylon 3D-printed acetabular models	Complex pelvic fractures, Perthes disease, DDH, OA with substantial bone loss	-	Changes in cup size, changes in surgical plan, comparison of different materials	Simulation with patient-specific 3D-printed models conferred superior clinical, logistical, and educational outcomes compared to CT and X-rays. Furthermore, it streamlined equipment selection and revealed potential complications
Xu et al. [25] 2015	Prospective	10 patients (14 hips)	3D-printed model for preoperative surgical simulation	DDH	23.1 ± 5.9 months (14–30)	HHS, LLD, cup coverage, hip center location, cup migration, cup sizing coincidence (ICC)	No surgical complications. Improvement of HHS *, no perceptible LLD, 83% average bone coverage, no cup migrations, all implants clinically and radiographically stable. Better sizing prediction in 3D planning compared to 2D planning *

DDH: developmental dysplasia of the hip; OA: osteoarthritis; HHS: Harris Hip Score; LLD: leg length discrepancy; CT: Computer Tomography; PLA: polylactic acid; ICC: interclass correlation coefficient; * = *p* < 0.05.

## Data Availability

Not applicable.
